# Degenerative changes of the canine cervical spine after discectomy procedures, an in vivo study

**DOI:** 10.1186/s12917-017-1105-5

**Published:** 2017-06-23

**Authors:** Peter Grunert, Yu Moriguchi, Brian P. Grossbard, Rodolfo J. Ricart Arbona, Lawrence J. Bonassar, Roger Härtl

**Affiliations:** 1 Department of Neurological Surgery, Weill Cornell Medicine, New York-Presbyterian Hospital, Weill Cornell Brain and Spine Institute|, 525 East 68th Street, Box 99, New York, NY 10065 USA; 2 Department of Neurological Surgery, Swedish Neuroscience Institute, Seattle, WA USA; 3 Department of Orthopedics and Neurosurgery VCA-Animal Specialty, Yonkers, NY USA; 4 Center of Comparative Medicine and Pathology, Memorial Sloan Kettering Cancer Center & Weill Cornell Medicine, New York City, NY USA; 5 Department of Biomedical Engineering, Cornell University, Ithaca, NY USA

**Keywords:** Canine spine, Discectomy, Degeneration, Disc herniation, Disc fenestration, Nucleus pulposus, Annulus fibrosus, Quantitative MRI

## Abstract

**Background:**

Discectomies are a common surgical treatment for disc herniations in the canine spine. However, the effect of these procedures on intervertebral disc tissue is not fully understood. The objective of this study was to assess degenerative changes of cervical spinal segments undergoing discectomy procedures, in vivo.

**Results:**

Discectomies led to a 60% drop in disc height and 24% drop in foraminal height. Segments did not fuse but showed osteophyte formation as well as endplate sclerosis. MR imaging revealed terminal degenerative changes with collapse of the disc space and loss of T2 signal intensity. The endplates showed degenerative type II Modic changes. Quantitative MR imaging revealed that over 95% of Nucleus Pulposus tissue was extracted and that the nuclear as well as overall disc hydration significantly decreased. Histology confirmed terminal degenerative changes with loss of NP tissue, loss of Annulus Fibrosus organization and loss of cartilage endplate tissue. The bony endplate displayed sclerotic changes.

**Conclusion:**

Discectomies lead to terminal degenerative changes. Therefore, these procedures should be indicated with caution specifically when performed for prophylactic purposes.

## Background

Discectomies are a commonly performed surgical procedures for the treatment of disc herniations in the canine spine [1, 2] and essentially describe an extraction of intervertebral disc (IVD) tissue out of the disc space. They are either performed independently using a ventral approach to treat simple disc protrusions without significant disc material extruding into the spinal canal [3] or in conjunction with a ventral slot procedure [1, 2]. Discectomies in the form of fenestration procedures are also performed prophylactically to reduce recurrence of disc herniation at the index segment or prevent further herniation at the adjacent levels above and below [4, 5]. The term discectomy and fenestration can

Discectomies comprise removal of the Nucleus Pulposus (NP) through a “window” or defect created in the Annulus Fibrosus (AF) via various approaches [6, 7]. The AF is only partially removed. Despite its crucial physiological function, such as maintaining disc height, and segmental stability [8–10] the extracted disc tissue is usually not replaced with an interbody device (e.g. cage, prosthesis or bone grafts) as done routinely in humans [11]. Removal of the disc and subsequent altered segmental functionality [8, 10, 12] could enhance degenerative changes [9, 13], which is why several authors advocate not to perform discectomies especially for prophylactic purposes [14, 15]. However, the correlation of discectomy procedures and segmental degenerative changes is somewhat hypothetical as it has not been studied in vivo. To fill this knowledge gap we studied radiological and histological changes of beagles undergoing ventral cervical discectomy procedures in vivo. Our goal was to evaluate direct postoperative changes within the first 16 weeks according to established diagnostic parameters. Specifically, we assessed qualitative and quantitative MR imaging to study morphological changes of spinal segments as well as residual Nucleus Pulposus size and hydration. Radiographs were performed ex vivo to assess for disc and foraminal height as well as probable fusion or osteophyte formation. Histological sections of discectomized segments were analyzed to evaluate degenerative changes of the Annulus Fibrosus, the Nucleus Pulposus, endplate cartilage and vertebral endplate bone.

## Methods

### Study design

All experimental procedures were reviewed and approved by the Institutional Animal Care and Use Committee at Weill Cornell Medicine (ethic’s protocol number: 2013–0110). Animals were housed in a facility accredited by the Association for the Assessment and Accreditation of Laboratory Animal Care (AAALAC) in compliance with applicable NY State, and Federal regulations.

This study is a subgroup analysis of an in vivo study evaluating tissue engineered intervertebral discs implanted in the beagle cervical spine [16] after undergoing a discectomy procedure. In this report we only include the control group of the main study which simply underwent a discectomy procedure without disc implantation.

Six skeletally mature male, sexually intact, purpose-bred beagles were included. They were obtained from Covance Research Products. At the time of surgery, the animals were 1 to 1.5 years old weighing between 15 and 25 kg.

All animals were assessed with magnetic resonance imaging (MRI) at 4, 8 and 16 weeks post surgery. After 16 weeks all animals were euthanized and specimens were harvested for ex vivo histology and radiographic analysis.

### Discectomy procedure

All animals were sedated with acepromazine hydromorphone cocktail given intramuscularly (IM). Anesthesia was induced with ketamine/midazolam cocktail given intravenously (IV), and maintained with a combination of IV fentanyl/lidocaine/ketamine and inhaled isoflurane. After endotracheal intubation, animals were placed in dorsal recumbency with the neck carefully extended supporting the nasal bridge. The surgical site was prepared by clipping hair and alternating chlorhexidine with alcohol solution three times. Each animal was preoperatively given acefazolin IV and repeated 2 h later during the surgical procedure.

A ventral midline incision was made from the base of the larynx to the sternum. The paired sternocephalicus and sternohyoideus muscles were separated by blunt dissection, exposing the trachea. Retractors were then positioned to retract the carotid sheath laterally and the trachea, and esophagus medially. The surgical level was identified by palpation of the prominent transverse process of C6. Small curved hemostats were used to separate the longus coli muscle overlying the ventral AF at C5-C6. The subsequent steps were carried out with a surgical microscope. After the ventral part of the AF has been incised (about 5 mm length) and resected with a scalpel and a Kerrison rongeur, the NP was completely extracted using a small tartar scraper, a 4–0 bone curette, and a Kerrison rongeur. Lastly, muscle fascia, subcutaneous tissue and skin were sutured.

### Qsualitative magnetic resonance imaging

We used a sagittal T2 sequence (TR = 2200 ms, TE = 66 ms, slice thickness = 2 mm) for qualitative MRI assessments (Siemens Tim TRIO 3 Tesla MRI Scanner, Erlangen, Germany). Images were obtained at week 8 and 16. Intervertebral disc (IVD) degenerative changes were evaluated according to the well established Pfirrmann grading scale [17], which outlines five grades of degeneration as defined by NP signal intensity, homogeneity and loss of disc height (Table 1). In addition, the canine specific grading scheme of Seiler et al. was used for evaluation. This classification takes NP signal intensity as well as AF morphology, possible disc herniation and spondylosis formation into account [18]. MRIs were further used to evaluate possible compression of nerve roots and the spinal cord.

**Table 1:** Pfirrmann MRI grading [17]

Grade	Structure	Distinction of Nucleus Pulposus	Signal intensity	Height of intervertebral disc
I	Homogeneous, bright white	Clear	Hyperintense, isointense to cerebrospinal fluid	Normal
II	Inhomogeneous with or without horizontal bands	Clear	Hyperintense, isointense to cerebrospinal fluid	Normal
III	Inhomogeneous, gray	Unclear	Intermediate	Normal to slightly decreased
IV	Inhomogeneous, gray to black	Lost	Intermediate to hypointense	Normal to moderately decreased
V	Inhomogeneous, black	Lost	Hypointense	Collapsed disc space

T1 flash sequences (TR = 3 ms, TE = 4.9 ms, slice thickness = 0.9 mm) with high bone contrasts were used to evaluate possible kyphotic or lordodic deformity changes of the operated segments. All discectomized discs were compared to adjacent levels control levels C6/C7.

### Quantitative magnetic resonance imaging

NP hydration and size were assessed with quantitative MRI using sagittal multislice multiecho pulse sequence (TR = 2000 ms, TE = 12 ms, NEX = 2, number of echoes = 12, echo spacing = 12 ms, slice thickness = 1 mm, and matrix size = 320 × 320, resolution: 125 μm × 125 μm × 1 mm). Images were obtained at week 4, 8 and 16.

NP hydration was assessed by its average T2 relaxation time, NP size by the amount of MRI NP voxels it is composed of.

The following algorithm previously developed by our group [19] was used for radiological segmentation of the NP:

A standard region of interest (ROI) measuring approximately 4 mm^2^ (comprising 300 voxels) was drawn within the center NP of the healthy disc proximal to the experimental segments. The average T2-relaxation time (T2-RT) of that ROI was measured, and this value minus 3 standard deviations was used to set a subtraction threshold for all voxels in that slice. Voxels with T2 values lower than the threshold were subsequently subtracted. As a result, only voxels with T2 values representing NP tissue remained in the disc space [19]. Subsequently, NP size was calculated by the amount of NP voxels it is composed of. The NP hydration was calculated by measuring the average T2 relaxation time of those NP voxels.

### Histological assessment

Animals were euthanized 16 weeks post-surgery by pentobarbital overdose IV. Cervical spines were harvested from C4-C7 and fixed using 10% neutralized formalin supplemented with 1% cetylpyridinium chloride (CPC). Spinal segments were decalcified, cut in a mid-sagittal plane and transferred to 75% ethanol. Subsequently segments were embedded in paraffin, cut to 5-μm thickness, and stained with Picrosirius Red, Safranin-O and Alcian Blue.

Degenerative changes were graded using the Bergknut classification which was established specifically for canine spine. The NP and AF are graded according to their staining characteristics, cell composition and endplate morphology [20, 21] (Table 2). Stained slides were observed under polarized light to evaluate the AF structure. Discectomized discs were compared to C6/C7 healthy control discs.

**Table 2:** Histological degenerative grading according to Bergknut et al. [20]

Morphology of annulus fibrosus (AF)	Chondrocyte metaplasia of AF	Tears and cleft formation	Chondrocyte proliferation of nucleus pulposus	Presence of notochordal cells in nucleus pulposus
0 Well-organized, half ring-shaped, collagen lamellae 1 Mild disorganized; some loss of half ring-shaped structure, most lamellar layer, still distinguishable (<25%) 2 Moderately disorganized; partly ruptured AF, loss of half ring-shaped structure (25–75%) 3 Completely ruptured AF; no or	0 No chondrocyte morphology, just spindle-shaped fibroblasts 1 Mild chondrocyte proliferation (i.e. limited to inner most AF layers) 2 Moderate chondrocyte proliferation (i.e. chondroid cells in up to half of the AF) 3 Marked chondrocyte proliferation (i.e. chondroid cells up to outer layers of the AF)	0 Absent 1 Rarely present 2 Present in intermediate amounts 3 Abundantly present 4 Scar/tissue defects	0 No proliferation 1 Increased chondrocyte-like cell density 2 Connection of two chondrocytes 3 Small size clones (i.e., several chondrocytes group together, i.e. 2–7 cells) 4 Moderate size clones (i.e. >8 cells) 5 Huge clones (i.e. >15 cells) 6 Scar/tissue defects	0 Abundantly present (>50%) 1 Present (1–50%) 2 Absent
Matrix staining of the nucleus pulposus with Alcian blue/Picrosirius red staining	Endplate morphology	New bone formation	Subchondral bone sclerosis	
0 Blue stain dominates 1 Mixture of blue and red staining 2 Red stain dominates	0 Regular thickness; homogeneous structure 1 Slightly irregular thickness 2 Moderately irregular thickness 3 Severely irregular thickness with interruption of the endplate	0 Absent 1 Minor new bone formation 2 Moderate amounts of new bone formation 3 Abundant new bone formation; tendency towards bridging/complete bridging	0 No sclerosis (<2 _ the thickness of the dorsal vertebral cortex) 1 Mild sclerosis (2–4 _ the thickness of the dorsal vertebral cortex) 2 Moderate sclerosis (>4 _ the thickness of the dorsal vertebral cortex) 3 Severe subchondral bone irregularities	

### Radiographic measurements

Radiographic images were performed in a digital radiographic cabinet (Faxitron). They were obtained 16 weeks post discectomy ex vivo directly after the cervical spine was collected for histological analysis. Images were used for disc height and foraminal height measurements. The IVD height was expressed as a disc height index, calculated by dividing disc height by adjacent vertebral body height on the basis of the modified method of Lu et al. [22]. Foraminal height was measured in pixels using the software pixel stick. Height of the discectomized segments is presented in percentage of the distal healthy adjacent segment (C6/C7). In contrast to quantitative MR imaging, we used the distal adjacent segment as a control for radiographic measurements. This is because two specimens were harvested at the C4/C5 disc space instead of the C4 vertebral body. Thus, this level could not be used for radiographic assessments in all specimens.

### Data analysis and statistics

For the analyses for continuous outcomes in disc height index, NP size, and NP hydration, generalized estimating equation (GEE) models were used to assess main effects and interaction factors of disc group and longitudinal assessment of time. Statistical analysis was performed with IBM SPSS Statistics 22 (SPSS, Chicago, IL, USA). *P* values <0.05 were considered statistically significant.

## Results

### Radiographic imaging

**Fig. 1:**
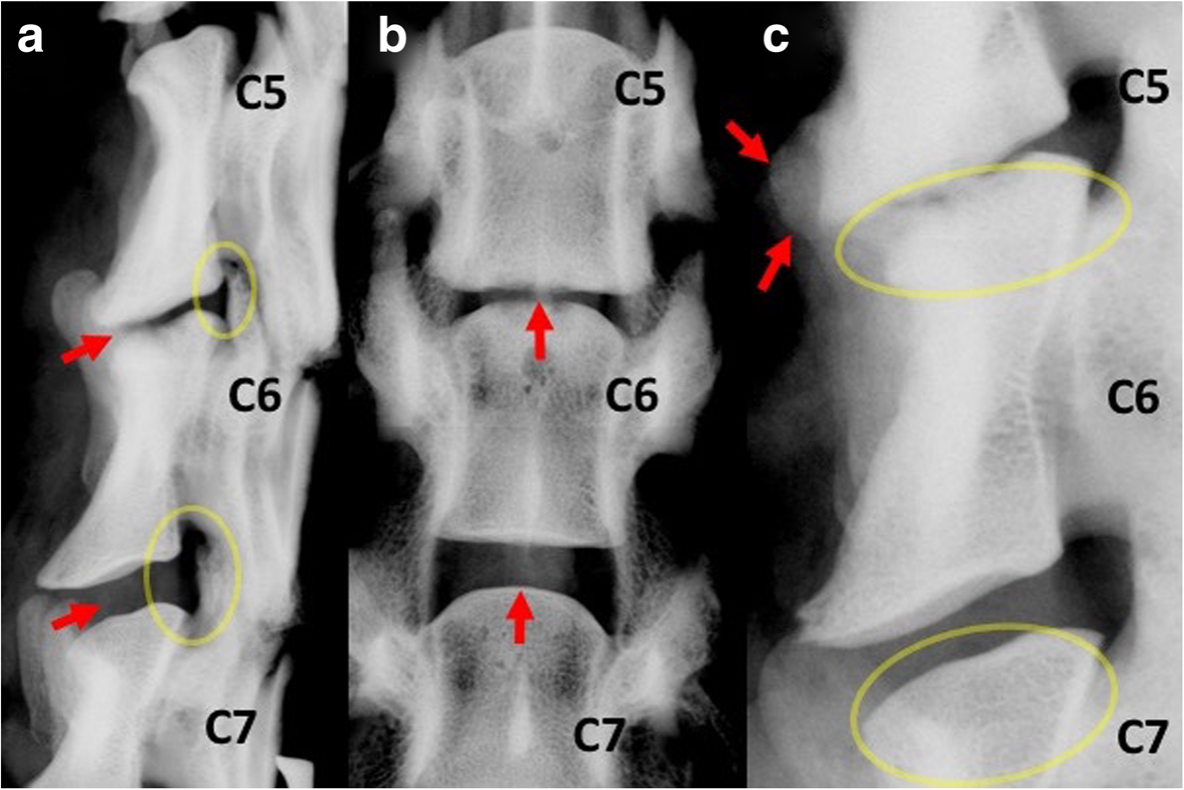
Radiograph of the lower cervical spine. **a** Lateral view, discectomy segment C5/C6 compared to adjacent healthy control C6/C7. *Red arrow* marks both disc spaces. The discectomy segment is almost completely collapsed. The neuroforamen of both segments are marked with a yellow ellipse. Loss in disc height subsequently lead to a loss of foraminal height. Both segments show no signs of kyphotic or lordotic deformity. **b** Ventro-dorsal view of the same specimen. *Red arrow* points to the disc space which is significantly narrowed at C5/C6. **c** Lateral view of different specimen. Bony endplates are marked with yellow ellipse. The healthy endplate at C6/C7 shows trabecular bone pattern. Sclerotic changes of the degenerated bony endplate at C5/C6. Endplate is more radio dense indicating increased cortical bone and decreased trabecular bone. *Red arrow* marks an osteophyte which formed ventrally at the C5 vertebral body

Healthy control discs showed no signs of degenerative changes on radiographs. Average DHI of healthy discs was 0.15 at week 16. Discectomized segments appeared collapsed at 16 weeks (Fig. 1a, b), showing a significantly lower DHI of 0.06 (<0.05) compared to adjacent control segments. This represents a 60% percent drop in disc height compared to adjacent controls.

Disc height reduction led to a subsequent decrease in neuroforaminal height (Fig. 1a, b).At 16 weeks the average height was 24% less in discectomized segments (C5/C6) compared to adjacent healthy controls at C6/C7.

Morphological changes included endplate sclerosis (Fig. 1c). Discectomized endplates became more radiopaque compared to healthy segments making the trabecular pattern of the endplate cancellous bone significantly less distinct. Discectomized segments also showed signs of spondylosis, ventrally at the the cranial vertebral body of the operated segment (Fig. 1c).

There was no significant angulation or kyphotic deformity present at operated segments. No fusion occurred according to radiographic images.

### Qualitative MR imaging

**Fig. 2:**
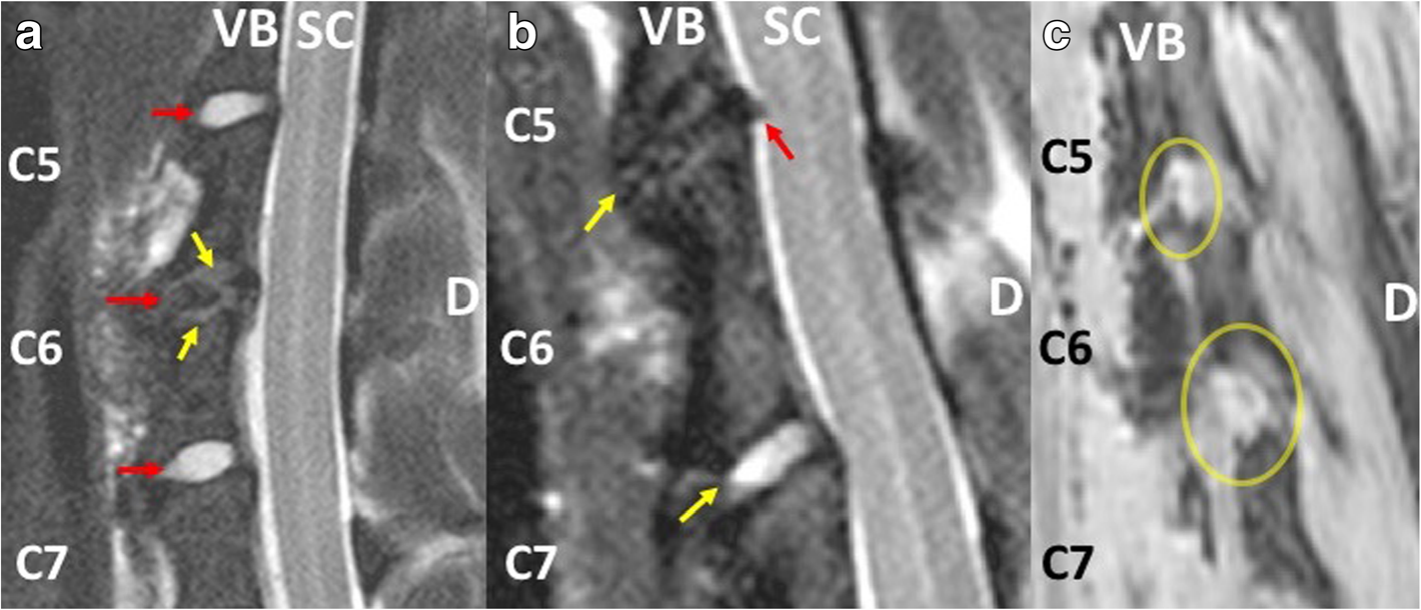
Sagittal T2 weighted MR images of the lower cervical spine. Right side of each slide is dorsal (D) **a** *Red arrows* point to the intervertebral discs between the vertebral bodies (VB) which are ventral to the spinal canal (SC). Healthy control discs at C6/C7 demonstrate a bright hyperintense, homogenous NP. The hyperintensity indicates normal NP tissue hydration. There is a clear border to the hypointense AF without loss of disc height. The endplates are hypointense. In contrast, the discectomy segment C5/C6 shows a black disc sign. There is complete loss of nuclear hyperintensity. The disc space is collapsed. The endplates (*yellow arrow*) are hyperintense which is indicative of type II Modic degenerative changes. **b** Different specimen. *Yellow arrows* point to disc space. *Red arrow* points to extruded disc at the discectomy segment. There is no significant spinal cord compression present. **c** Lateral view to the neuroforamen (*marked by yellow ellipse*). The C5/C6 discectomy segment shows a narrower foramen compared to the healthy C6/C7 segment. However, the nerve root (hypointense structure within foramen) appears not to be compressed at the discectomy segment

After 16 weeks, healthy adjacent discs (C6/C7) showed no signs of degenerative changes. Five discs displayed a Pfirrmann degeneration grade I, indicating a completely healthy disc with a homogenous bright hyperintense Nucleus Pulposus on T2 weighted images (Fig. 2a). The hypointense AF showed a clear border to the nucleus. No bulging of the AF was present. One specimen showed a Pfirrmann grade of II which represents a healthy disc with a less homogenous bright NP showing a nuclear cleft, which is a horizontal gray band representing fibrous tissue dissecting the nucleus [18]. According to the canine specific Seiler grading, adjacent discs (C6/C7) of five animals were evenly graded as I indicating completely healthy discs. One specimen was graded as II for displaying a nuclear cleft.

At week 16 all discectomized IVDs (C5/C6) showed a Pfirrmann grade of V, representing terminal degenerative changes with a completely collapsed discs space and “black disc” sign (hypointense on T2 weighted images) (Fig. 2a). According to the canine specific Seiler grading all C5/C6 discs were evenly graded as V indicative of progressed degenerative changes.

In contrast to healthy endplates (C6/C7) which appear hypointense (dark) on T2 weighted images, endplates in discectomized discs became hyper intense which is indicative of type II Modic changes.

Disc protrusions without significant spinal cord or nerve root compression were also visible (Fig. 2b).

The collapsed disc space also led to a decrease in size of the neuroforamen which narrowed the space for the exiting nerve root (Fig. 2c). However, a clear sign of nerve root impingement was not visible.

### Quantitative MR imaging

Healthy nuclei showed an average size of 361 NP voxels. Average T2 relaxation time (correlating to NP hydration) was 372 ms. Both did not significantly change over 16 weeks.

**Fig. 3:**
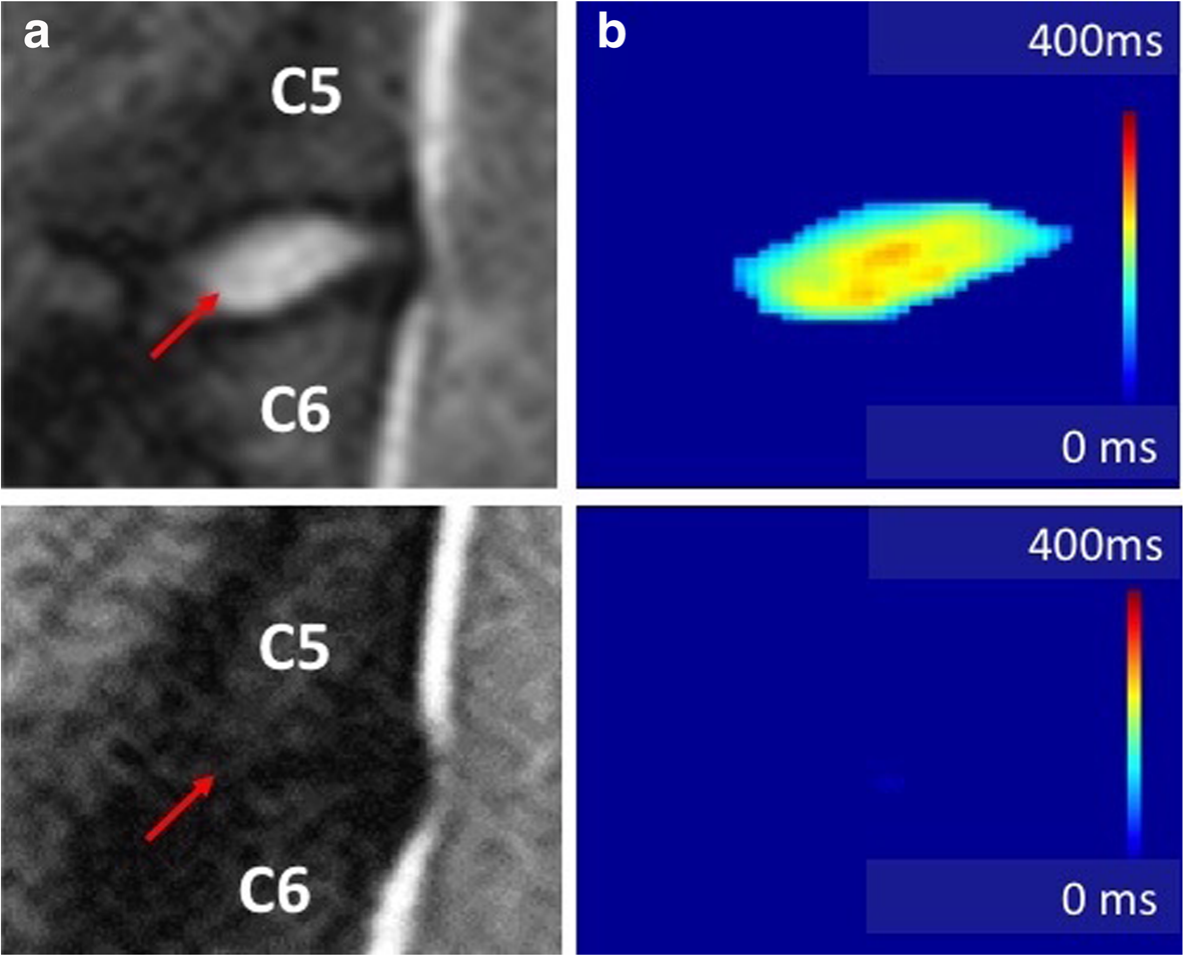
Display of quantitative MR imaging for nuclear size and hydration measurements. **a** Sagittal T2 weighted MR image of a healthy (top) and degenerated disc (bottom). **b** Corresponding T2 relaxation time of the NP displayed as a heat map. Red colors represent high T2 relaxation time (high tissue hydration), *blue colors* low T2 times (low tissue hydration). All MRI voxels not representing NP tissue according to their T2 relaxation time were subtracted leaving only MR voxels representing nuclear tissue in the slide. The size of the nucleus was measured by the amount of NP voxels it was composed of. The degenerated disc on the bottom shows no residual NP voxels indicating that there is no nuclear tissue present on that slide

Discectomized discs showed a significant decrease in NP size compared to healthy disc with an average NP voxel count of 16 (*p* < 0.05) (4 specimen were 0) by week 4 and 2,25 (*p* < 0.05) (4 specimen were 0) by week 16 (Fig. 3a, b). Compared to healthy discs, hydration of the residual NP decreased by 4 weeks as T2 relaxation time decreased to an average of 113 ms (*p* < 0.05). The average T2 relaxation time on week 16 was further reduced to an average of 42 ms (*p* < 0.05).

### High bone contrast MR imaging

No significant kyphotic or lordotic deformity was observed after discectomy procedures at 16 weeks.

### Histology

**Fig. 4:**
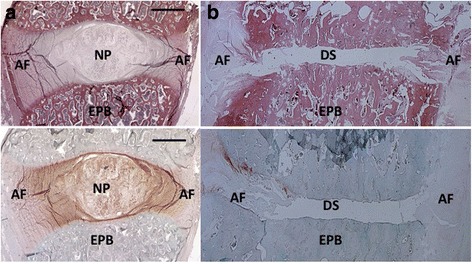
Histology sections of explanted cervical segments (×2 magnification). **a** Healthy discs. Top Picrosirius red stain (stains for collagen), bottom Safranin-O stain (stains for proteoglycans). Both discs show a well organized AF composed of lamellar aligned collagen fibers which stain intense for Picrosirius *red*. There is a clear border to the NP which appears as a homogenous structure. It stains with Safranin-O indicating proteoglycan rich matrix. The cartilaginous endplate (*black arrow*) forms a border between the NP to the endplate bone (EPB). It stains intensively with Safranin-O indicating high proteoglycan content. **b** Discectomy discs. Top Picrosirius red, bottom Safranin-O stain. The annulus is disrupted and disorganized on both sides. Annular Picrosirius red staining is less intense. There is no NP tissue left in the disc space (DS). The cartilaginous endplate completely disappeared. The Endplate bone is sclerotic indicated by dense cortical bone structure and decreased trabecular bone. The collapse of the disc space is visible on histological sections

After 16 weeks, healthy C6/C7 discs showed no signs of degenerative changes. The AF was composed of lamellar parallel aligned fibers and spindle shaped fibroblasts and appeared well organized (Fig. 4a). The Nucleus Pulposus was predominantly composed of notochordal cells. The nucleus stained intensively with Safranin O for proteoglycans. The endplates had a regular homogenous thickness and cartilage structure with chondrocytes embedded in a proteoglycan rich matrix (indicated by intense Safranin O staining) (Fig. 4a). The average Bergknut degeneration grading score was 0 out of 24 indicating no degenerative changes.

**Fig. 5:**
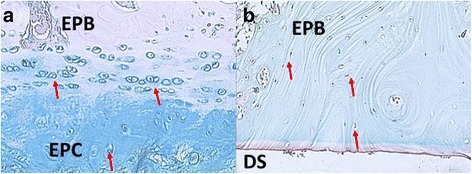
Endplate bone Alcian blue stain (stains for proteoglycans). **a** Healthy disc ×8 magnification. The endplate cartilage (EPC) consists of chondrocytes (*red arrows*) embedded in a proteoglycan rich matrix (*blue stain on alcian blue*). The cartilage endplate borders to the endplate bone (EPB). **b** Discectomy disc ×4 magnification. The bony endplate with its cortical bone structure and osteoclasts (*red arrows*) borders directly to the disc space. There is no cartilage endplate in between

**Fig. 6:**
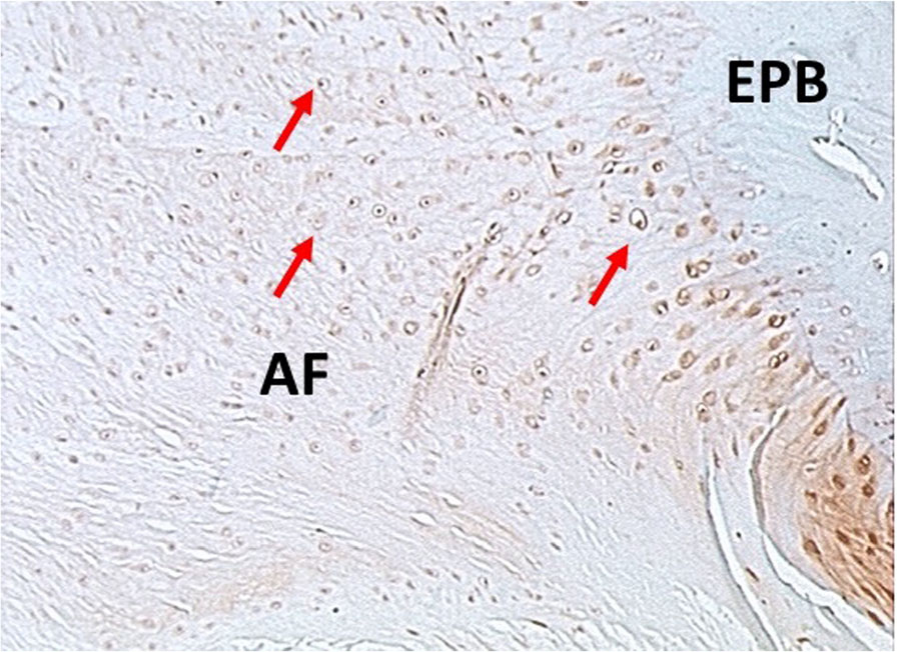
Safranin-O stain of the Annulus Fibrosus which is attached to the endplate bone (EPB). Chondrocytes (*red arrows*) are infiltrating the annulus replacing fibroblasts, a degenerative process called chondrocytic metaplasia

Discectomized C5/C6 discs in contrast showed progressed signs of degeneration after 16 weeks. The AF was partially ruptured; cleft formation was present (Fig. 4b). The AF appeared less organized, displaying alterations of its lamellar structure under polarized light (Fig. 5). The AF cell composition changed from fibroblasts to chondrocytes, a typical degenerative change described as chondrocyte metaplasia (Fig. 6). All specimen showed no residual NP tissue in the disc space (Fig. 4b).

**Fig. 7:**
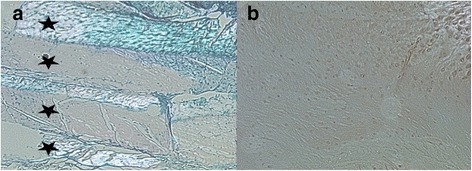
Alcian *Blue* stained slides of the AF under polarized light (×4). **a** Healthy disc, the AF is organized in a multilamellar structure. The lamellae (*black star*) are composed of parallel aligned fibers and are therefore birefringent (bright) under polarized light. They alternate in their orientation resulting in alternating birefringence. **b** Dorsal annulus of a discectomy disc. Tissue lost its multilamellar organization. The Annular fibers lost their organization and parallel alignment indicated by lost in birefringent behavior under polarized light

Both endplates were thinned and disrupted. The cartilage endplate completely disappeared (Fig. 7) and bony endplate (subchondral bone) displayed sclerotic changes (Fig. 4b). There was no new bone formation present on histological sections. The average degeneration grade according to the Bergknut classification was 19.4 out of 24 indicating terminal degenerative changes.

## Discussion

Disc herniation is a common neurological condition in dogs, specifically in small and chondrodystrophoid breeds such as the beagle [23]. Discectomy procedures are commonly performed independently or in conjunction with a laminectomy or ventral slot approach for this condition.

They are also performed prophylactically as fenestration procedures. Due to their technical similarity, the term discectomy and fenestration can be used synonymously in this manuscript.

The goal of this study was to evaluate the degenerative effects of discectomies in the canine spine.

We could show that IVDs undergoing discectomies displayed terminal signs of radiographic, and histological degeneration according to established grading scales. These alterations occurred relatively early after a 16 week follow up. Degeneration affected all three components of the IVD: the NP, AF and endplates.

### NP changes

The NP plays a crucial role for segmental biomechanical functionality. It has a relatively high proteoglycan content leading to a high water binding capability [24]. The water content allows the NP to establish a hydrostatic pressure, which separates both endplates thereby maintaining disc height [24]. The relatively high water content is also responsible for the nuclear viscoelastic mechanical properties which facilitates its important damping quality [25].

According to MR based NP voxel count measurements, over 95% of the nucleus were removed from the discectomy procedure by week 16. The morphological appearance of “black discs” on T2 weighted images confirmed these quantitative assessments. Loss of NP T2 signal intensity represents loss of tissue hydration [26, 27]. It is a critical degenerative parameter according to the MR Pfirrmann grading, indicating terminal degenerative changes. Histological sections confirmed the almost complete loss of NP tissue, which is also a critical degenerative parameter [28].

### AF changes

The AF, with its complex multilamellar fiber structure, is crucial for maintaining segmental stability by opposing axial rotation as well as lateral bending [29]. It works in tandem with the NP by opposing nuclear hydrostatic pressure facilitating maintenance of disc height.

Compared to the NP, there are less typical radiological signs for the AF to describe degenerative changes such as signal alterations. However, MR imaging showed bulging of the AF towards the spinal canal which is an important degenerative parameter according to canine specific Seiler MR grading [18]. Annular bulging most likely developed due to the loss of disc height and the resulting outwards displacement of annular fibers. Reduced disc height as well as reduced damping properties due to NP loss leads to redistribution of axial load and shear forces to the AF which results in structural changes [30, 31]. Histological sections of the AF in our study revealed early signs of these structural changes with cleft formation, reduced organization of lamellar structure and chondrocyte metaplasia.

### Cartilage endplate

The cartilage endplate, with its high proteoglycan content, allows for high solute diffusivity that facilitates nutrition supply to the avascular NP [32, 33]. The endplate also has a mechanical function, as it absorbs hydrostatic pressure from the NP and works as a barrier to prevent bulging of the NP into the endplate [34].

Similar to the AF, there are no established radiographic signs to describe cartilage endplate degeneration. There are however indirect MR signs of endplate pathology. Modic changes, specifically type II, are described as increased T2 signal intensity in the vertebral body bone tissue adjacent to the endplate [34]. The histopathological correlate of type II Modic changes are disruption of the end plate with signs of an active inflammatory reaction [34]. Both increased endplate T2 signal intensity as well as disruption of the endplate on histological sections were seen in discectomized discs in our study. Modic changes have shown to correlate with back pain in humans [35]; a potential clinical relevance has so far not been studied in the canine spine. Endplate disruption can impact its physiological function. Disrupted endplates demonstrated an impaired ability to contain hydrostatic pressure from the NP which could result in disc height loss [36]. Endplate discontinuity has also shown to induce degenerative changes of IVDs, likely by impacting nutrient transport to nuclear cells [34, 37, 38].

It is unclear if endplate damage and thinning resulted from a post discectomy degenerative process or from the procedure itself. While it is possible this was the result of iatrogenic damage, it is unlikely since we took great care to not damage the endplates during discectomy procedures.

### Comparison to literature

To our knowledge, this is the first in vivo study evaluating degenerative changes following ventral discectomy procedures performed with a standard surgical technique. Shores et al. studied degenerative changes of canine spinal segments after disc fenestration using a needle puncture technique with partial Nucleus Pulposus aspiration. Similar to our study, there was radiographic evidence of disc height narrowing 24 weeks post surgery. Histological sections showed no inflammation, dissolution or fibrotic changes on the residual NP. The only histological changes described were vascular and fibrous tissue invasion of the punctured AF [13]. Compared to our study, it appears that smaller puncture defects with retained NP tissue induce less degenerative changes than incising the AF with complete NP removal. However, similar studies in rabbit and rat demonstrated that puncture defects lead to terminal degenerative changes over time [16, 39]. They have also shown that the AF has a very low intrinsic healing capability [40, 41] and that residual NP tissue can herniate through annular needle puncture defects [16] over time.

While we did not evaluate mechanical alterations of discectomized discs, others have conducted mechanical studies on canine cervical cadavers undergoing C5/C6 discectomy procedure. They demonstrated a significant increase in flexion/extension range of motion post surgery indicating that these procedures may have a destabilizing effect of the involved segment [8, 12].

### Clinical considerations

We could demonstrate that discectomy procedures as described are effective in completely removing NP tissue which makes herniations at prophylactically treated segments less likely to occur.

This corroborates studies showing a lower reherniation rate in animals treated with prophylactic fenestrations [42, 43].

However, discectomy procedures induced degenerative changes in a relatively short period of time. The most significant change was loss of disc height which led to a subsequent loss of neuroforaminal height. Reduced neuroforaminal height can lead to nerve root impingement which subsequently can cause radicular symptoms such as referred pain, muscle weakness or loss of sensation. Discectomy procedures also lead to protrusion of the AF into the spinal canal which could in certain cases cause symptomatic spinal cord compression. However, neither disc protrusion nor neuroforaminal stenosis led to significant impingement of neural structures after 16 weeks. There was also no deformity present on MR or radiographic imaging. It has to be kept in mind that 16 weeks is a short term follow up and that spinal degenerative changes such as spinal canal stenosis or deformity have a tendency to occur and progress over time.

Considering the degenerative effect demonstrated in our study, prophylactic discectomies in the form of fenestration procedures should be carefully considered specifically when they involve segments without significant preoperative radiographic signs of degeneration. Besides degenerative changes, fenestration procedures have further reported disadvantages such as longer surgery times, higher morbidity with increased postoperative pain and deficits as well as complications such as seroma formation or life threatening hemorrhages into the thorax cavity potentially leading to postoperative death [43].

### Limitations

The main limitation of this study is the relative short term follow up. Degenerative changes described could progress over time. Another limitation is that healthy discs underwent discectomies whereas in clinical practice treated discs often already display signs of degeneration, which could alter histological appearance. Another limitation is that only one level underwent a discectomy. Prophylactic discectomies are often performed on several levels, which could worsen spinal malalignment.

## Conclusion

Discectomies lead to significant degenerative changes of the affected discs according to histological, MR and radiographic assessments. Degenerative changes involved all three components of the IVD: the nucleus, Annulus Fibrosus and endplate. Therefore, discectomy procedures should be considered carefully especially when performed for prophylactic purposes.

## Abbreviations

AF: Annulus Fibrosus
EPB: Endplate bone
EPC: Endplate cartilage
IM: Intramuscular
IV: Intravenous
IVD: Intervertebral disc
MRI: Magnetic resonance imaging
NP: Nucleus Pulposus
ROI: Region of Interest
T2-RT: T2 Relaxation time
